# A gasless laparoscopic technique of wide excision for gastric gastrointestinal stromal tumor versus open method

**DOI:** 10.1186/1477-7819-11-44

**Published:** 2013-02-25

**Authors:** Po-Chu Lee, Peng-Sheng Lai, Ching-Yao Yang, Chiung-Nien Chen, I-Rue Lai, Ming-Tsan Lin

**Affiliations:** 1 Department of General Surgery, National Taiwan University Hospital, No.7 Chung-Shan South Road, Taipei 100, Taiwan; 2 Department of Trauma, National Taiwan University Hospital, No.7 Chung-Shan South Road, Taipei 100, Taiwan; 3 Department of General Surgery, National Taiwan University Hospital, Yun-Lin Branch, No.579, Sec. 2, Yunlin Rd., Douliu City, Yunlin County, 640, Taiwan

**Keywords:** Gastrointestinal stromal tumors, Laparoscopic surgery, Surgical instruments

## Abstract

**Background:**

Traditional open surgery for gastrointestinal stromal tumors (GIST) requires a long incision. Moreover, the gas-filling laparoscopic technique used in GIST surgery still has its limitations. Therefore, we developed a gasless laparoscopic (GL) surgery for GIST and compared it with traditional open surgery.

**Methods:**

Between October 2007 and September 2009, 62 GIST patients in the National Taiwan University Hospital received wide excisions. Of these 62 patients, 30 underwent the new procedure (GL group) and 32 had open surgery (OS group). Preoperative and postoperative clinicopathologic characteristics were compared between the groups.

**Results:**

There were no significant differences in preoperative characteristics or blood loss. However, the days to first flatus, postoperative hospital stay, wound length, white blood cell count at postoperative day one, and peak daily body temperature were all significantly improved in the GL group. Usage of postoperative analgesia on postoperative days one to five was also significantly lower in the GL group.

**Conclusions:**

Wide-excision laparoscopy for gastric GIST can be performed more safely, more effectively, and with faster postoperative recovery using the gasless technique as compared with the open method. We, therefore, recommend this new surgical technique, which hybridizes the advantages of both the traditional open method and pure laparoscopic surgery.

## Background

Surgical resection is the standard treatment for gastrointestinal stromal tumors (GIST), but the surgical methods require improvement. GIST, the most common mesenchymal tumor of the gastrointestinal tract, are divided into benign, intermediate, malignant, and those with malignant potential [1-4]. Lymph node dissection for malignant stromal tumor is optional. Among GIST patients, no difference in survival between systemic lymph node dissection and non-dissection groups was observed [5]. Wide excision for small malignant gastric stromal tumors without lymph node dissection is thought to be an acceptable treatment protocol [6-10]. Therefore, minimal access is potentially applicable and beneficial for GIST surgery. A traditional laparotomy may require a larger wound and a longer postoperative recovery period, whereas laparoscopic surgery may have advantages over open surgery that include early recovery of bowel function, early hospital discharge, and decreased pain [11,12]. However, there are still difficulties and risks related to the use of the gas-filling laparoscopic method for GIST in some areas, such as the posterior wall or the cardia area of the stomach [13], and this surgery is often lengthy.

Based on the gasless laparoscopy procedure proposed by Hyodo *et al*. [14], we developed a new technique using gasless laparoscopy with abdominal wall-lifting and applied this in wide-excision surgery for GIST [15]. Herein, we describe the use of this innovative gasless laparoscopic environment for GIST surgery and compare it with traditional open surgery with regard to therapeutic potential, feasibility and effectiveness.

## Methods

### Patients and data recorded

This retrospective study retrieved data from the surgical database of the National Taiwan University Hospital of cases coded as gastric gastrointestinal tumors postoperatively. Sixty-two patients underwent wide excision of the stomach for removal of the lesion from October 2007 to September 2009. The medical records of all the patients were retrospectively reviewed. No patients were excluded. All patients provided informed consent for the surgical procedure and for the report to be published. The choice of gasless laparoscopy-assisted minimally invasive surgery (GL group) or traditional open surgery for tumor excision (OS group) was based on the surgeon’s preference, while no significant difference was found in the years of surgical experience among the surgeons. Informed consent was obtained from every patient, and the possibility of open conversion was explained to the patients who underwent gasless laparoscopy-assisted surgery. Preoperative gastrofiberoscopy, endoscopic ultrasonography [16] and computed tomography [17] were performed prior to surgery to localize the tumor and confirm the diagnosis. If the tumor was smaller than 2 cm, it was clipped with localization nails around the circumference. Preoperative imaging revealed no lymph node or liver metastasis in any of the cases in this study. Postoperative care for both groups followed the same protocol: all patients fasted postoperatively until flatus, and prophylactic antibiotics (cefazolin) were administered after surgery.

All patient data were obtained from surgical records, surgical pathology reports, and the attending surgeon’s records. The following patient characteristics were collected: age, gender, body mass index (BMI), American Society of Anesthesiology (ASA) score, tumor size (longest diameter), tumor location, length of wound, days to first flatus, postoperative hospitalization duration, operation duration, estimated blood loss, and the leukocyte count on postoperative day one. Morphine usage and highest body temperature were recorded on postoperative days one to five.

### Second generation of the new surgical procedure of gasless laparoscopic wide excision

**Figure 1:**
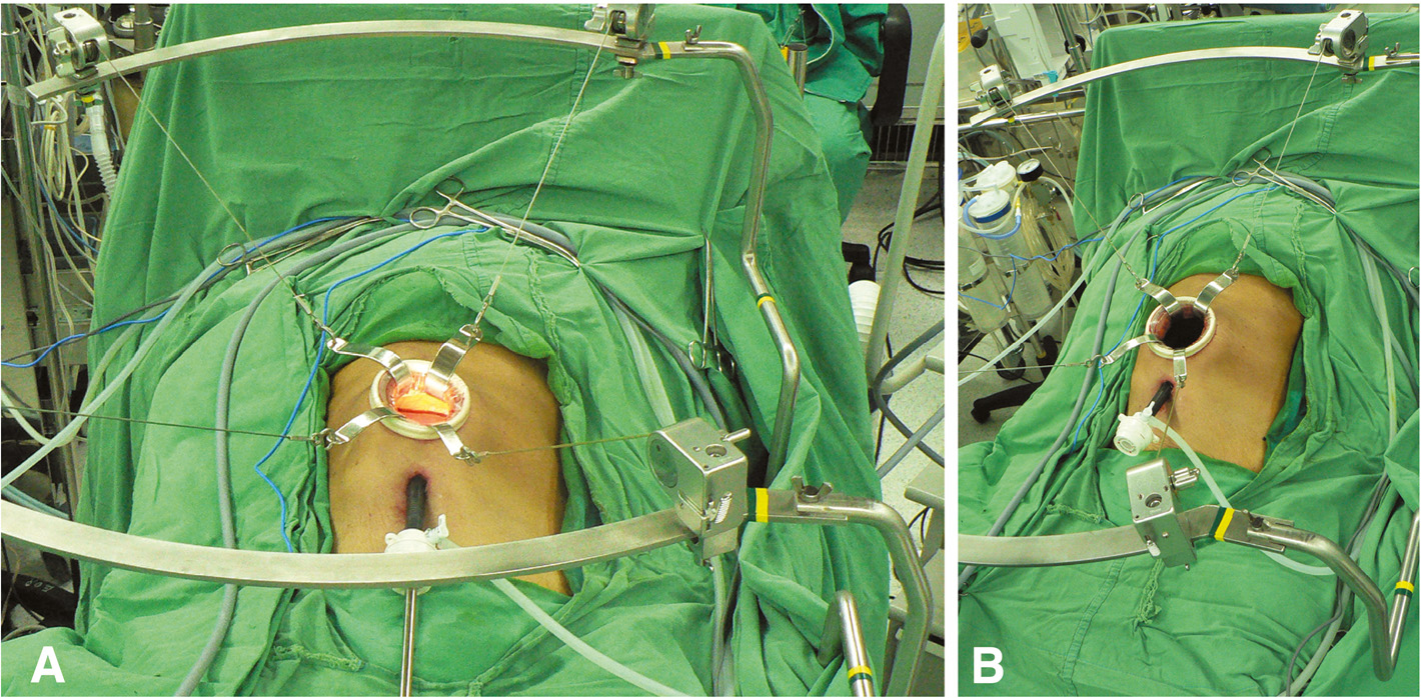
(A) and (B) Mini-laparotomy (5 cm) at the upper midline.

**Figure 2:**
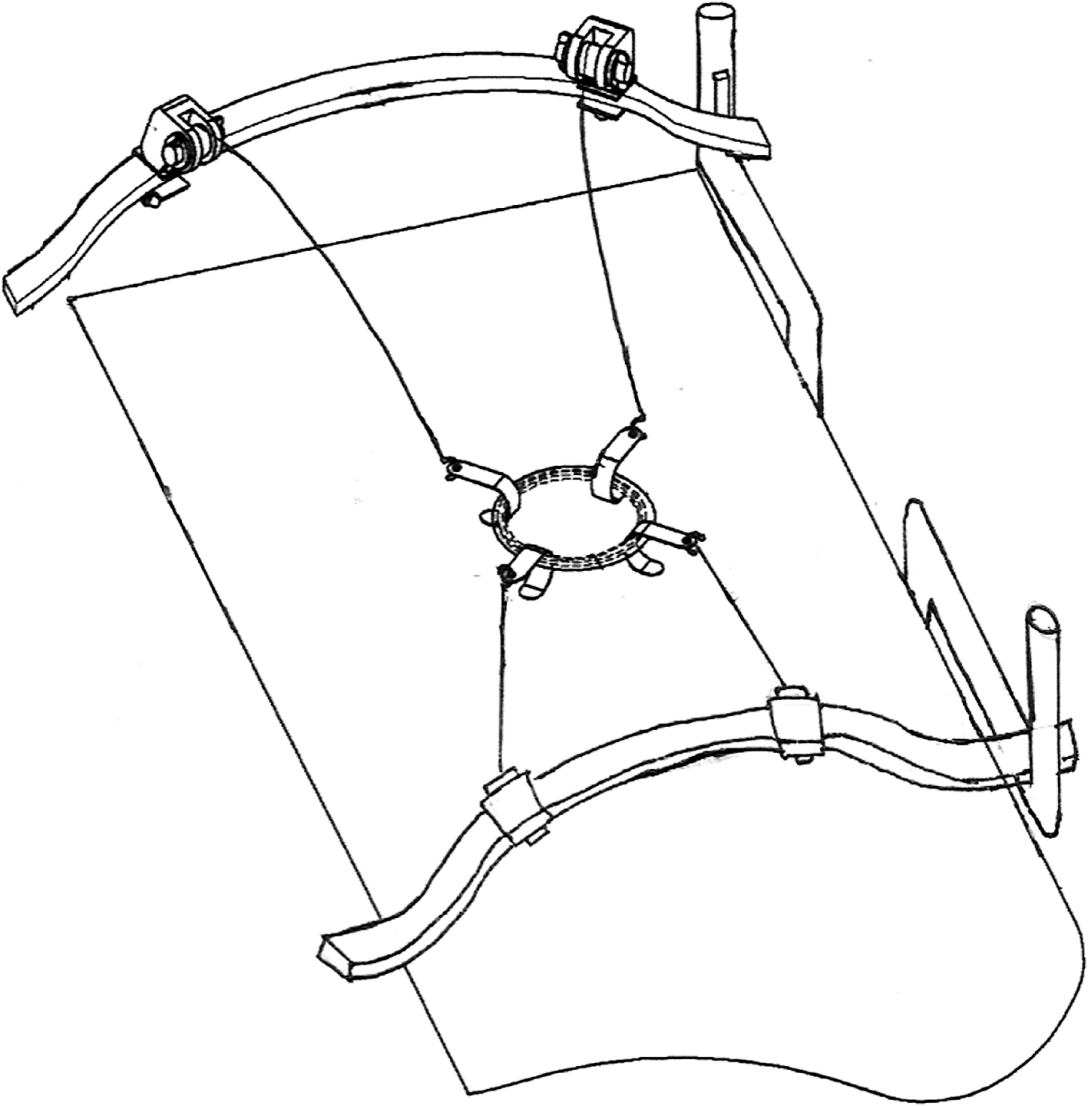
Instrument design.

As in the technique of gasless laparoscopy-assisted subtotal gastrectomy used in our previous study [15], a 3 to 5-cm minilaparotomy was made in the upper midline. A wound protector was used to cover the minilaparotomy wound to facilitate retraction and to avoid contamination or tumor cell implantation. We lifted up the abdominal wall using our newly-designed second generation self-sustained retractors (Figure 1A and B). Three working ports (5 or 10 mm) were created at the bilateral subcostal and para-umbilical areas. Three-dimensional imaging was achieved promptly and easily by simultaneous direct vision and laparoscopic vision: direct vision of the tumor was achieved by looking through the minilaparotomy wound, while a laparoscope was inserted through one of the ports or the mini-laparotomy to achieve full vision of the tumor and to identify the relative location of the tumor with regard to the surrounding vital organs. The liver and peritoneal cavity were examined using a laparoscope to determine whether any metastases were present. Tactile examination of the tumor was possible under this gasless environment and the tumor or the location clip was palpated using one or two fingers through the mini-laparotomy wound to clarify its position and extension. The gastrocolic ligament, lesser omentum and perigastric tissue were divided using a Ligasure auto-coagulator (Tyco, Valleylab, CO, USA) or a Harmonic scalpel (Ethicon, Cincinnati, OH, USA). If necessary, traditional instruments such as forceps and suction can also be used via the mini-laparotomy wound. After fully freeing the stomach and the tumor, a wide excision was made intracorporeally through the port using a stapler (EndoGIA, Tyco) under laparoscopic observation, and an endoretractor was used to retract the liver, colon and adjacent tissue. A schematic diagram of the instrument design is shown in Figure 2.

If the tumor was close to the esophagogastric (EG) junction, a wide excision was made to protect the EG junction by looping it with a nelaton catheter. The tumor was then completely excised, and anastomosis was performed using an endostapler (EndoGIA, Tyco) completely intracorporeally under direct and laparoscopic observation. The resected specimen was isolated in a plastic bag and retrieved through the mini-laparotomy wound that had been made at the beginning of the operation.

If the tumor was too large, the specimen was extracted using a mechanical crushing method with a ring clamp from a thick-walled specimen bag. Meticulous hemostasis was confirmed and a rubber drain was inserted through a previous working port. The mini-laparotomy wound was then closed, completing the operation. The other group underwent the standard operation of OS for GIST via a traditional laparotomy. Total excision of the tumor was undertaken using a traditional electric knife, and gastroplasty was performed with two layers of interrupted sutures.

### Statistical analysis

Demographic and clinical data including age, BMI, ASA score, tumor size (longest diameter), wound length, duration before return of normal bowel movements, postoperative hospitalization duration, operation duration, estimated blood loss, and the leukocyte count on day one were tabulated as the mean ± standard deviation.

Statistical analysis was performed using SPSS 17, and the non-paired *t*-test and Fisher’s exact test were used to compare the two study groups (GL versus OS) with respect to all continuous or ordinal variables. A *P* value <0.05 was considered significant.

## Results

The GIST patients in our study consisted of 20 men and 42 women with a mean age of 62.5 years (range, 22 to 85 years). The GL group included 30 patients, while 32 patients in the OS group underwent traditional OS for wide excision. In the GL group, seven tumors were located in the cardia area, six in the fundus area, ten in the body area, and seven in the antrum area, while in the OS group, nine tumors were located in the cardia area, seven in the fundus area, nine in the body area, and seven in the antrum area.

As shown in Table 1, there were no significant differences in age, gender, BMI, ASA score, or tumor size (longest diameter) between the two groups. The GL patients’ ages ranged from 22 to 71 years, whereas the OS patients ranged from 42 to 85 years of age. The BMI of the GL group was 24.7, compared to 24.3 in the OS group. The average ASA score, as scored by the anesthesiologist, was 2.3 for the GL group and 2.1 for the OS group.

**Table 1:** Demographic data of the patients in this study

	**GL group**	**OS group**	
	**(number = 30)**	**(number = 32)**	***P *value**
Age (years)	62 ± 12.5	62 ± 11.8	0.97
Sex ratio (M:F)	8:22	12:20	0.28
BMI (kg/m^2^)	24.7 ± 2.5	24.3 ± 2.9	0.59
ASA score	2.3 ± 0.5	2.1 ± 0.3	0.06
Tumor size^a ^(cm)	5.84 ± 1.92	7.0 ± 2.3	0.645
Location: cardiac: fundus: body: antrum	7:6:10:7	9:7:9:7	

^a^Tumors were measured after resection as A × B × C cm (for example, A = 8 cm, B = 4 cm, C = 3 cm), and tumor size was recorded as the longer length (A: 8 cm). It was, therefore, possible that, on occasion, the tumor was larger than the incision wound. Values are mean ± standard deviation. *ASA *American society of anesthesiology; *GL *gasless laparoscopy; *OS* traditional open surgery.

Operative and postoperative recovery data indicated that the wound length, duration to first flatus, postoperative hospitalization days, and postoperative white blood cell count on day one differed significantly between the groups (Table 2). The wound length was significantly smaller in the GL group (5.1 cm versus 10.1 cm); first flatus was detected earlier in the GL group (2.5 days versus 4.0 days); postoperative hospitalization was significantly shorter in the GL group (7.1 versus10.7 days); and the postoperative day one white blood cell count was lower in the GL group (8,770/mm^3^ versus 11,470/mm^3^).

**Table 2:** Perioperative data of the patients in this study

	**GL group**	**OS group**	
	**(number = 30)**	**(number = 32)**	***P *value**
Length of wound (cm)	5.1 ± 0.8	10.1 ± 3.4	<0.001
Days to oral intake	2.5 ± 0.7	4.0 ± 0.9	<0.001
Postoperative hospital stay (days)	7.1 ± 1.9	10.7 ± 2.3	<0.001
Postoperative day one WBC (per mm^3^)	8,770 ± 2,160	11,470 ± 2,042	<0.001
Operation time (minutes )	116.6 ± 26.1	119.6 ± 48.5	0.19
Estimated blood loss (mL)	58.5 ± 30.1	82.7 ± 80.4	0.12

*WBC *white blood cell count.

**Figure 3:**
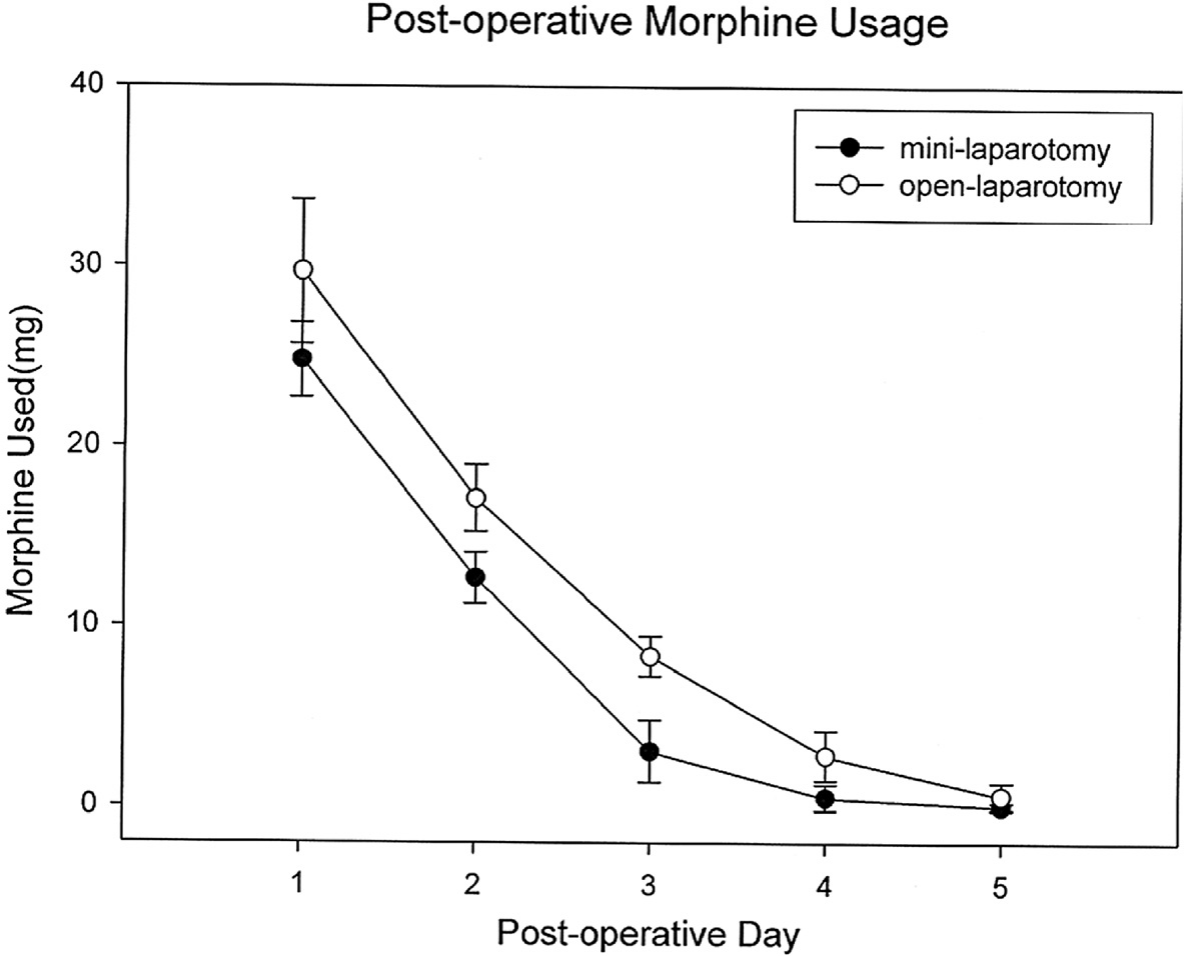
**Postoperative morphine usage was lowest in the mini-laparotomy group (GL group), with a significant difference between groups in morphine usage on postoperative days one to five (*P *< 0.05).**

**Figure 4:**
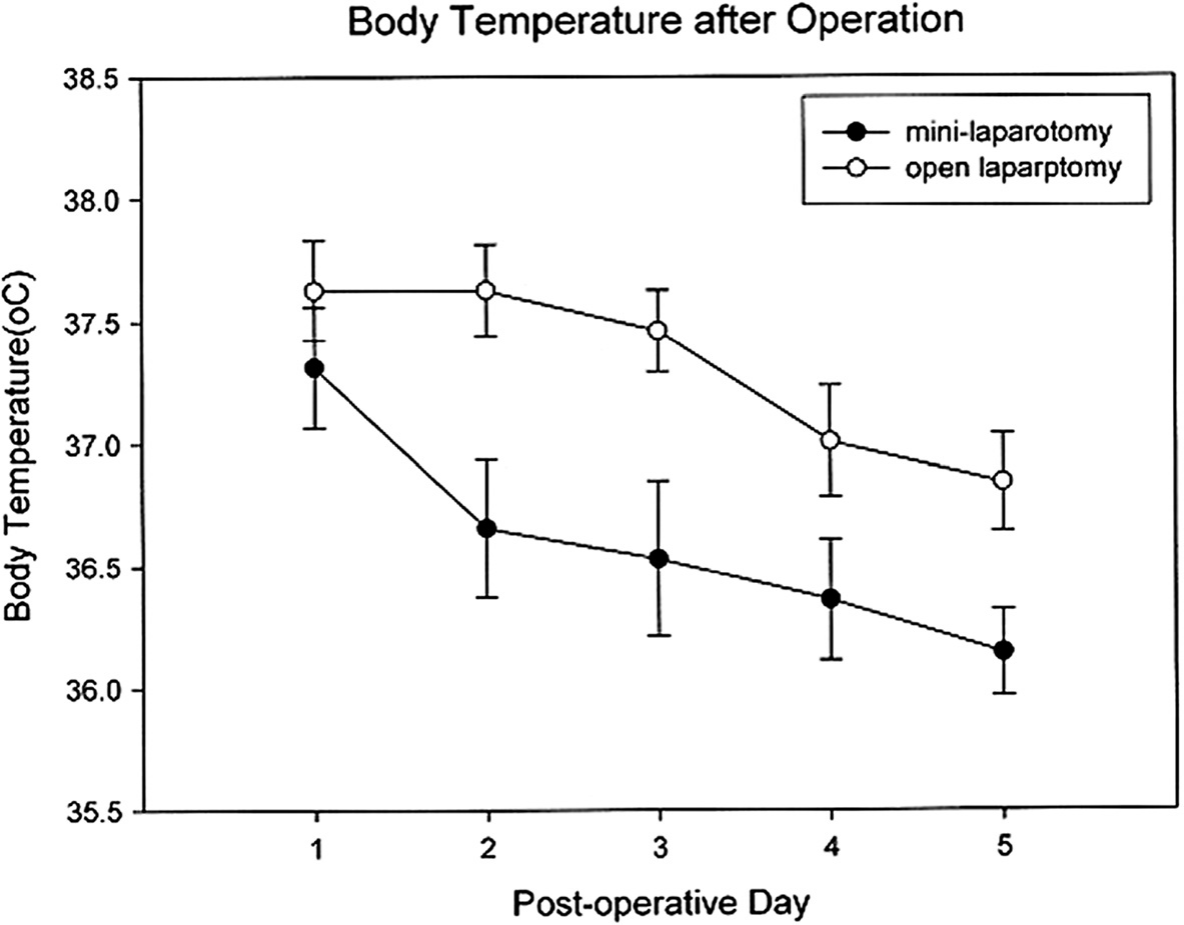
The postoperative daily highest temperature curve shows significantly lower body temperatures on postoperative days one to five in the mini-laparotomy group (GL group).

However, there were no significant differences in terms of operation duration or estimated blood loss between the GL and OS groups (116.6 minutes versus 119.6 minutes and 58.5 ml versus 82.7 ml). All patients received pethidine hydrochloride via intramuscular injection for pain control, and the dose of analgesic administered on days one to five was higher in the OS group (Figure 3, *P* <0.05). The highest body temperature observed on postoperative days one to five was also higher in the OS group (Figure 4, *P* <0.05).

## Discussion

GIST are uncommon tumors, but are the most common mesenchymal tumors of the gastrointestinal tract [18]. Many reports have shown that it is not necessary to remove lymph nodes in patients with GIST [5-10]. Therefore, an effort is needed to develop a method of minimal access for GIST surgery.

We previously developed a procedure for laparoscopy-assisted radical subtotal gastrectomy, which achieved good results [15]. The second generation of self-sustained retractors separated the previous circular stent into two lunar-shaped stents. Using this newly designed equipment, the space for the surgeon and assistants is widened and the visual field is also enlarged in gasless surgery. This new technique is a safe method in which an approach can be made via any position on the stomach, and recovery may be faster as compared with the OS method. As no randomized study has been carried out on the methods of stomach GIST surgery previously, in this study we applied this new surgical procedure in GIST patients to validate the advantages of laparoscopy over conventional surgery.

Some studies have suggested that laparoscopic surgery, when applied to GIST, can shorten the postoperative hospitalization duration, lessen cytokine reactions, and produce a better cosmetic result [11,12,19]. Our GL gastrectomy takes advantage of the benefits of laparoscopic surgery, including a smaller wound, fewer analgesics and faster recovery; it is also easy to perform, as no new complicated instruments are involved.

Yano and colleagues [20] reported that hand-assisted laparoscopic surgery may be a more suitable procedure for patients with a large GIST because it offers adequate traction for the resection, which is often obscured from the laparoscopic view in large GISTs. The wound in our gasless setting is smaller than that occurring with the use of the hand-assisted device. The gasless setting is considered as a bridge between OS and laparoscopic surgery. The advantages of the technique over hand-assisted techniques are a smaller wound, no CO_2_ needed, and a greater similarity to total laparoscopic surgery. For small-size GISTs, the use of intraoperative gastric endoscopy is effective and can obviate the need for tactile sensation. However, endoscopic resection is time consuming. In contrast, in the gasless setting, smaller tumors can be palpated directly. There is no need to set up an intraoperative endoscopy in gasless surgery, which can reduce the operation time.

Controlling massive bleeding in a gas-filled state is a critical issue. Adequate suction is not always possible. Some studies have shown that cardiopulmonary function is compromised in older GIST patients and the risk of port-site metastasis is increased under the gas-filling laparoscopy system [21-24]. In contrast to the limitations of gas-filling laparoscopic GIST resection, our new gasless technique maintains the advantages of both traditional laparotomy and gas-filling laparoscopy.

We found that if the vessels are engorged or not coagulated, they can be tied securely using a knot pusher inserted through the mini-laparotomy wound [25]. With the assistance of the laparoscopic system, vision was increased, which allowed more delicate dissection of vessels. This may explain why the blood loss was lower in the GL group (58.5 ml versus 82.7 ml), although this difference was not significant.

For the GL group, the operation duration was not significantly longer than for the OS group (116.6 minutes versus 119.6 minutes), a result similar to those of other studies comparing the use of laparoscopy-assisted gastrectomy and conventional open gastrectomy for the surgical treatment of early gastric cancer [26-28].

Further, the GL method can provide direct vision through the mini-laparotomy wound in addition to the laparoscopic view, which helped to achieve three-dimensional vision easily and to avoid accidental injury owing to the dead space involved in laparoscopy. No postoperative complications occurred in the GL group, nor was there massive bleeding or accidental injury to other vital organs, which would have required conversion to traditional OS.

Our new surgical procedure, GL gastrectomy, has the same advantages as gas-filling laparoscopic surgery, such as less opiate analgesia required, a faster recovery and a shorter hospital stay [27-30]. We found that the time to recovery of normal bowel movements and the length of hospital stay were shorter in the GL group (2.5 versus 4.0 days and 7.1 versus 10.7 days, respectively). Furthermore, the dose of analgesics given on the first postoperative day was significantly lower in the GL group (Figure 3).

Hayashi *et al*. [29] reported decreased serum IL-6 and C-reactive protein and a lower white blood cell count, all suggesting less inflammation, in patients who undergo laparoscopic-assisted distal gastrectomy. Our GL patients experienced less inflammation because of the smaller incision and lesser bowel manipulation, resulting in lower levels of release of cytokines and other thermoregulatory compounds and lower body temperatures. In addition, less pain and the absence of pneumo-peritoneum may result in a lower level of fever secondary to pulmonary atelectasis. We found the white blood cell count to be significantly lower in the GL group (8,770/mm^3^ versus 11,470/mm^3^), and the peak daily body temperature was also lower on postoperative days one to five in the GL group (Figure 4). This technique may be of particular benefit in terms of enabling older patients to return to a normal level of activity within a few days and reducing the risk of surgical complications.

## Conclusion

The novel traction instrument arrangement designed by our team has many advantages over OS for the treatment of stomach GIST, but there has been no previous randomized study to compare the two treatment options. We, therefore, treated GIST patients using our newly-developed GL wide-excision procedure and evaluated the results in comparison with those of traditional OS. The use of our technologically-innovative surgical instruments enabled minimally-invasive GIST surgery to be performed safely, and this method was effectively applied to treat gastric GIST at any location, resulting in no major postoperative complications, allowing a more rapid recovery, and reducing the postoperative hospitalization period as compared with traditional laparotomy.

## Abbreviations

ASA: American society of anesthesiology; BMI: Body mass index; GIST: Gastrointestinal stromal tumors; GL: Gasless laparoscopy; IL: Interleukin; OS: Open surgery.

## Competing interests

The authors declare that they have no competing interests.

## Authors’ contributions

We declare that all the listed authors participated actively in the study and all meet the requirements of authorship. CYY and PCL designed the study and wrote the protocol; CNC, IRL and MTL performed research/study; CCY undertook the statistical analysis; and PSL wrote the first draft of the manuscript. All authors read and approved the final manuscript.
